# Surface-modified deproteinized human demineralized tooth matrix for bone regeneration: physicochemical characterization and osteoblast cell biocompatibility

**DOI:** 10.1093/rb/rbae030

**Published:** 2024-03-21

**Authors:** Natwara Chokwattananuwat, Srisurang Suttapreyasri

**Affiliations:** Department of Oral and Maxillofacial Surgery, Faculty of Dentistry, Prince of Songkla University, Songkhla, Thailand; Department of Oral and Maxillofacial Surgery, Faculty of Dentistry, Prince of Songkla University, Songkhla, Thailand

**Keywords:** acid-modified dpDTM, biocompatibility, bone graft, collagen-modified dpDTM, deproteinized human tooth matrix, tooth-derived bone graft

## Abstract

Tooth presents an intriguing option as a bone graft due to its compositional similarity to bone. However, the deproteinized human demineralized tooth matrix (dpDTM), developed to overcome the limited availability of autologous tooth grafts, has suboptimal pore size and surface roughness. This study aimed to fabricate a surface-modified dpDTM using acid etching and collagen coating, followed by *in vitro* evaluation of physicochemical and biological properties. The dpDTM was modified into two protocols: Acid-modified dpDTM (A-dpDTM) and collagen-modified dpDTM (C-dpDTM). Results demonstrated that A-dpDTM and C-dpDTM had increased pore sizes and rougher surfaces compared to dpDTM. Collagen immobilization was evidenced by nitrogen presence exclusively in C-dpDTM. All groups had a Ca/P molar ratio of 1.67 and hydroxyapatite as the sole constituent, with 65–67% crystallinity. Degradation rates significantly increased to 30% and 20% for C-dpDTM and A-dpDTM, respectively, compared to 10% for dpDTM after 120 days. Cumulative collagen release of C-dpDTM on Day 30 was 45.16 µg/ml. Osteoblasts attachment and proliferation were enhanced on all scaffolds, especially C-dpDTM, which displayed the highest proliferation and differentiation rates. In conclusion, surface modified of dpDTM, including A-dpDTM and C-dpDTM, significantly enhances bioactivity by altering surface properties and promoting osteoblast activity, thereby demonstrating promise for bone regeneration applications.

## Graphical Abstract

**Figure:**
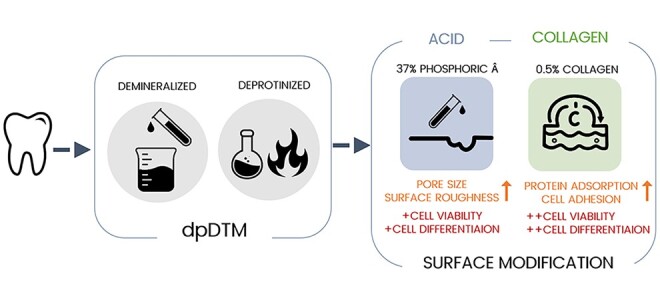

## Introduction

Large bone defects result from trauma, post-extraction, infection, disease and tumor resection affecting patient health and quality of life [1]. Bone tissue engineering is a novel approach to promote bone repair and regeneration. Bone tissue engineering consists of three components: cells, biomimetic agent such as growth factors, and biomaterial scaffold. Biomaterial scaffolds, in bone tissue engineering, are important structure for promoting cell adhesion and proliferation [2]. Ideal bone scaffold should have several key features including: biocompatibility, biodegradability, bioactivity, suitable porosity, good mechanical properties, and mimic to natural bone extracellular matrix (ECM) [3].

Four main categories of bone grafts are available for use in medical procedures: autogenous, allografts, xenografts, and alloplasts. Autogenous bone from iliac, calvarial or intraoral donor site has been considered for many years as the gold standard for bone graft due to its osteogenesis, osteoinduction, and osteoconduction [4–8]. Autologous tooth bone graft materials are an intriguing option to use as a bone graft substitute, as the chemical composition of teeth is similar to bones, both being composed of organic and inorganic materials. The main inorganic material of tooth is calcium phosphate including hydroxyapatite (HA), beta-tricalcium phosphate, amorphous calcium phosphate and octacalciumphosphate [9]. These inorganic materials contribute to the physicochemical and mechanical strength of the bone. Consequently, numerous studies have explored and implemented the use of autologous tooth bone grafts in various dental surgeries, such as alveolar ridge preservation, alveolar ridge augmentation, and maxillary sinus augmentation. However, one limitation of autologous tooth bone grafts is their restricted quantity. Allogenic tooth grafts have emerged as a potential alternative in dental surgery. The deproteinized human demineralized tooth matrix (dpDTM) was developed as allograft material, offering advantages over autologous tooth bone grafts, including an unlimited supply and convenient storage. The deproteinization process removes organic materials from extracted teeth through either heat or chemical treatment, thereby reducing immunogenicity [10]. Heat treatment breaks down the hydrogen bond of polypeptide chain [11] while chemical treatment precipitates proteins by altering the pH [12]. Previous research [13] has demonstrated that dpDTM treated with NaOH/thermal method eliminated immunogenic proteins while retaining the inorganic calcium phosphate composition. dpDTM demonstrates biocompatibility, low immunologic response, and non-toxicity, making it a promising option for bone graft substitution materials in dental surgery. This includes procedures such as alveolar ridge preservation for delayed implant placement (manuscript submitted for publication) and maxillary sinus augmentation (manuscript submitted for publication). However, surface modification may further enhance its properties [13].

Surface modification is a crucial process aimed at enhancing the appropriate physicochemical and mechanical properties of biomaterials to influence specific cellular and tissue responses, particularly in the context of bone regeneration [3, 14].

Surface modification methods can be broadly categorized into two techniques: subtractive modification and additive modification. Subtractive modification entails procedures such as surface correction through acid etching, sandblasting, laser methods, anodizing, and sandblasting with large-grit acid-etched [3].

Subtractive modifications are extensively employed to create porous and rough surfaces, influencing cellular responses, including cell adhesion, proliferation, and differentiation [15, 16]. Phosphoric acid etching is a commonly utilized surface treatment method for dentin and other materials like dental implants. The application of acid etching to dental implants enhances their biological properties, ultimately fostering osseointegration and bone formation. Additionally, phosphoric acid etching in dentin dissolves the peritubular dentin, increasing the pore size of dentinal tubules, which, in turn, stimulates cell adhesion and proliferation, contributing to bone formation [17, 18].

Additive modification of biomaterials includes techniques that add structures to the surface including processes such as coating and plasma spray [19]. Coating is one of the most common additive modification techniques. Coating material can be categorized into organic, inorganic, and composite materials. Furthermore, polymers have found extensive use in surface modification, and they are classified into two primary groups: natural polymers and synthetic polymers.

Collagen, a bioactive natural polymer, predominates as an essential component in biological tissues. Notably, type I collagen, representing over 90% of the organic mass of bone [20], is characterized by a triple-helical structure with hydrogen bonds. Type I collagen has proven effective in surface modification of biomaterials by introducing additional bioadhesive motifs, such as asparagine-glycine-glutamate-alanine or glycine-phenylalanine-hydroxyproline-glutamate-arginine. These motifs are assumed to serve as binding ligands for the α2β1 integrins of osteoblasts, resulting in enhanced osteoblast and fibroblast adhesion [21–23].

Moreover, collagen is the principal protein constituent of the ECM, facilitating efficient nutrient transportation. It possesses several advantageous properties, including excellent biocompatibility, minimal immunogenicity, low toxicity, an interconnected porous structure, and biodegradability. These attributes promote cell migration, adhesion, and differentiation [24–26]. Nevertheless, a notable limitation of collagen lies in its relatively low mechanical strength, restricting its direct clinical applications [27, 28]. To overcome this limitation, collagen has been combined with other materials to mimic the bone’s ECM, aiming to develop composite biomaterials that yield superior bone regeneration outcomes [25].

While previous studies have extensively documented surface modification of dentin surface for providing surface micro-retention for dental fillings [9, 17, 29, 30], there is a notable absence of evidence concerning the surface modification of tooth graft prepared for bone substitution material. Based on these considerations, the present study focuses on the development of novel composite scaffolds for bone regeneration using the surface modification of dpDTM through acid etching and collagen incorporation, to alleviate the problems encountered when using dpDTM in its original form. The physicochemical characterization was evaluated, measuring surface characteristics, elemental contents, phase composition, Ca/P ratio, crystallinity percentage, degradability, and release of collagen. Furthermore, the osteoblast cell biocompatibility tests were assessed to demonstrate favorable cell–material interactions implying physiological responses in terms of viability, proliferation, and differentiation.

## Materials and methods

The study was approved by the Human Research Ethic Committee of the Faculty of Dentistry, Prince of Songkla University (EC6412-076).

### Fabrication of surface-modified dpDTM (A-dpDTM, C-dpDTM)

The dpDTM were prepared according to a previous study [13]. Briefly, caries-free premolars and third molars extracted as part of orthodontic treatment were collected from the Oral Surgery Clinic, Faculty of Dentistry, Prince of Songkla University. All patients participating in this study provided written informed consent for the use of their extracted teeth in the experiment. Any tooth with pathology or structural anomalies was excluded from the study. The teeth were meticulously cleaned, then divided into crown and root sections, with the pulp and periodontal tissue removed mechanically.

The mineralized tooth particles were achieved through a mixer ball mill machine (Mixer Mill M301, Retsch GmbH, Haan, Germany) and sieved with apertures of 500 and 1000 μm (Endecotts, London, UK) to select particles within the 500–1000 μm size range. For the fabrication of the demineralized tooth matrix (DTM), a partially DTM was created by treating it with 0.5 M HCl for 3 h at 25°C, followed by thorough washing with double-distilled water. The dpDTM was prepared through a chemical/thermal process. Specifically, 5 g of DTM was immersed in 50 ml of 1 N NaOH at 37°C for 14 days on a rocking platform, with daily solvent replacement. Then, the thermal treatment was executed by annealing the DTM at 300°C with a heating rate of 30°C/min and held for 10 min. Finally, the tooth particles underwent bleaching using 30% H_2_O_2_ for 10 min at 40°C.

#### Fabrication of acid-modified dpDTM (A-dpDTM)

The dpDTM was etched with 37.5% phosphoric acid (H_3_PO_4_) for 15 s then was rinsed with distilled water several times.

#### Fabrication of collagen-modified dpDTM (C-dpDTM)

The acid-modified dpDTM (A-dpDTM) was immersed in 0.5% types I bovine collagen (Gibco™, Thermo Fisher Scientific, Inc., Waltham, MA, USA). After 2 h, samples were removed from the solution and rinsed several times in 1% aqueous acetic acid, to remove excess adsorbed collagen. After rinsing, samples were immersed in water containing 0.25% 1-ethyl-3-(3-dimethyl aminopropyl) carbodiimide (EDC) and 0.25% *N*-hydroxysuccinimide (NHS) and kept overnight in this coupling solution for three nights. After coupling, all samples were carefully rinsed with 1% aqueous acetic acid and water, then dried under a hood.

### Physicochemical characterization

Physicochemical characterization was performed to investigate the surface macro/micro architecture, crystallinity, and composition of dpDTM and modified dpDTM (three samples/group).

#### Scanning electron microscope and scanning electron microscope with energy-dispersive X-ray analysis

The surface macro/microstructure was analyzed by scanning electron microscope (SEM; SU3900, Hitashi, Japan). The samples were dried, sputter-coated with gold thin film and viewed with SEM system. The electron micrographs were obtained at 100×, 1000×, 1500×, 5000×, and 25 000× magnifications. The pore size distribution histograms were plotted in Qtiplot (Bucuresti, Romania) using processed SEM micrographs from Image J software (NIH, Bethesda, MD, USA).

The quantitative of element component on the sample’s surface was examined by SEM with energy-dispersive X-ray (SEM-EDX) analysis (SEM; SU3900, Hitashi, Japan).

#### Brunauer–Emmett Teller analysis

The surface area was examined using Brunauer–Emmett Teller (BET) (Micromeritics ASAP2460, Micromeritics Instrument Corp, Atlanta, GA, USA). The analyses were determined by nitrogen gas adsorption–desorption at boiling point of liquid nitrogen.

#### X-ray fluorescence spectroscopy and Fourier transform infrared spectroscopy

The elemental component in sample was analyzed by X-ray fluorescence spectroscopy (XRF) (PW 2400, Phillips, Eindhoven, Netherlands). The stoichiometric Ca/P ratios were calculated using the following formula [31]:

$$\mathrm{Ca}\;(\mathrm{mol})/P\;(\mathrm{mol})\;=\;\frac{\mathrm{Ca}\;(\mathrm{wt}\%)}{40.08\;(g/\mathrm{mol})}/\frac{P\;(\mathrm{wt}\%)}{30.97\;(g/\mathrm{mol})}$$

The protein contents and the functional group of material in sample were determined by Fourier transform infrared spectroscopy (FTIR) (Bruker VERTEX 70, Bruker Optik Inc, Ettlingen, Germany).

#### X-ray diffractometer and fluorescence microscope

The calcium phosphate phase and the crystallinity structure (peak area/total area) were evaluated by X-ray diffractometer (XRD) (X’Pert MPD, Philips, Eindhoven, Netherlands) with Cu Ka radiation (40 kVp and 30 mA).

All samples were labeled with the ATTO-TAG™ CBQCA Derivatization Reagent (CBQCA; 3-(4-carboxybenzoyl)quinoline-2-carboxaldehyde) (Thermo Fisher Scientific, Inc.) following the manufacturer’s protocol. In brief, CBQCA reagent solutions were prepared by dissolving the reagent (3 mg/ml) in methanol (0.1 mM) and potassium cyanide (10 mM). After that, all samples were immersed in solution and incubated at room temperature for 2 h. All samples were identified by confocal laser scanning microscopy (CLFM; Zeiss LMS 800, USA).

### *In vitro* degradation and collagen releasing

*In vitro* degradability of the sample was analyzed in simulated body fluid (SBF) as described elsewhere [32, 33]. Briefly, the samples were immersed in SBF, which were prepared in accordance with Kokubo’s study [34], over 60 days for determining the degradation in terms of weight loss (%). Briefly, the sample was weighed (W0) before immersing in 1 ml of 50 mM Tris-HCl solution pH 7.4 at 37°C for 120 days. The sample was collected on 1, 3, 7, 14, 30, 60, 90, and 120 days for assessment of weight change (*n* = 3 per group/time point). At each time point, the sample was blotted with sterile paper and freeze-dried (Labogene ApS, Germany) for 24 h before weighted (*Wt*). Weight loss of the sample was calculated using the following equation.

$$\%\mathrm{weight}\;\mathrm{loss}=100\times \frac{W0-Wt}{W0}$$

The dissolution of collagen was analyzed using the Collagen Assay Kit (Sigma-Aldrich, Schnelldorf, Germany). The samples of each group were immersed in 5 ml of SBF, which were prepared in accordance with Kokubo’s study [34], and placed in a shaker at 37°C and 90 rpm. On Days 1, 3, 5, 7, 14, 21, and 30, the scaffolds were moved into the next wells, and fresh solutions were added. The solution from each previous well was collected for released collagen using the Collagen Assay Kit, following the manufacturer's protocol. In brief, 20 µl of protein solution was mixed and incubated with 30 µl of Master Reaction Mix for 60 min at 37°C. Afterwards, 40 µl of dye reagent was added and incubated for 10 min at 37°C. Then, 8 µl of developer was added and incubated for 10 min at 37°C. The fluorescence of the solution was measured at *λ*ex/em = 375/465 nm. The levels of OD were compared with a standard curve to calculate the concentration of collagen releasing.

### *In vitro* bioactivity characterization

#### Preparation of scaffold

All samples were fabricated into scaffold by using polyvinyl alcohol (PVA) hydrogel solution as binding materials. The dpDTM, A-dpDTM, and C-dpDTM were synthesized according to a previous study with modification [13]. Briefly, 5 wt% PVA solution was prepared; 300 mg of dpDTM, A-dpDTM, and C-dpDTM into 200 µl of 5% PVA solution, mixed thoroughly and then perfused into a container (diameter of 10 mm and height 3 mm). The dpDTM, A-dpDTM, and C-dpDTM hydrogel was subjected to freeze–thaw cycles at temperatures of –20 ± 2°C (freezing) and 25 ± 2°C (thawing) for 3 days. The scaffolds were sterilized using ethylene oxide gas and were maintained under sterile condition before use.

#### Cell culture

Osteoblast cell lines (MC3T3-E1 subclone 4, ATCC, USA) were grown in the basal medium (α-MEM supplemented with 10% fetal bovine serum and 1% penicillin–streptomycin) and cultivated in 5% CO_2_ at 37°C until reaching confluence and then subculturing was performed. Before the cell seeding, the scaffolds were pre-incubated in a cell culture medium at 37°C for 24 h. MC3T3-E1 osteoblasts cells (5 × 10^5^ cells/scaffold) were dropped into each scaffold and incubated at 37°C for 2 h. Scaffold seeded with MC3T3-E1 was cultured in 500 µl of basal medium for proliferation with a replacement of medium every 2 days.

#### Cell morphology

The cells were seeded on the scaffolds at 5 × 10^5^ cells/scaffold and cultured as previously described (*n* = 5 per time point). At Days 1, 3, and 7, scaffold was dried and coated with gold–palladium then SEM (SU3900) was used to visualize cell adhesion and morphology.

#### Cell viability

Cell viability of osteoblast cells was assessed using Live/Dead^TM^ Viabililty/Cytotoxicity kit for mammalian cells (Thermo Fisher Scientific, Inc.) on Days 1, 3, and 7, following manufacture’s protocol. In brief, Live green solution (Component A) and dead red solution (Component B) were transferred into phosphate buffered saline (PBS) to create staining solution, which was then added into samples and incubated at room temperature for 15 min. The stained cells (*n* = 3) were observed and imaged using CLFM. Six images in each sample were randomly chosen, and live cells (green-stained) and dead cell (red-stained) were counted using Image J software. Cell viability was calculated by dividing the total number of cells by number of green-stained cells.

PrestoBlue cell viability (Invitrogen) was used to determine cell viability on the scaffold on Days 1, 3, and 7 (*n* = 5 per time point). Briefly, 100 ml of PrestoBlue^TM^ Reagent (Invitrogen Corporation, San Diego, USA) was added to 900 ml culture medium directly to cells and incubated at 37°C for 2 h according to the manufacturer’s protocols. Then, 200 µl of the supernatant was collected for measurement. The OD was then measured at 570 nm using microplate reader (Thermo Scientific, Waltham, MA, USA). The levels of OD were compared with a standard curve to infer the amounts of the cells.

#### Cell differentiation

For evaluate cell differentiation, osteoblast cell lines (MC3T3-E1 subclone 4, ATCC, USA) was grown in the osteogenic medium (basal medium with 50 µg ascorbic acid, 1 µM dexamethasone and 10 µM β-glycerophosphate) and cultivated in 5% CO_2_ at 37°C until reaching confluence and then subculturing was performed. Before the cell seeding, the scaffolds were pre-incubated in a cell culture medium at 37°C for 24 h. MC3T3-E1 osteoblasts cells (5 × 10^5^ cells/scaffold) were dropped into each scaffold and incubated at 37°C for 2 h. Scaffold seeded with MC3T3-E1 was cultured in 500 µl of basal medium with a replacement of medium every 2 days.

#### Alkaline phosphatase assay

Alkaline phosphatase (ALP) was measured using ALP activity staining kit (Human Diagnostics Worldwide, Magdeburg, Germany), in accordance with manufacturer’s instructions. Briefly, on Days 1, 7, 14, and 21 thereafter, cells were seeded on a scaffold that has been grown in the osteogenic medium (basal medium with 50 µg ascorbic acid, 1 µM dexamethasone and 10 µM β-glycerophosphate). The scaffold was rinsed with 1× PBS and added 0.1% Triton-X solution, then freeze–thawed three times. Afterward, substrate solution was measured using an ALP activity staining kit. The OD was then measured at 570 nm using a microplate reader. The levels of OD were compared with a standard curve to infer the amounts of the cells.

#### Osteocalcin assay

Osteocalcin (OCN) activity was measured using mouse OCN Elisa Kit (Elabscience, USA), in accordance with manufacturer’s instructions. Briefly, on Days 1, 7, 14, and 21 thereafter, cells were seeded on a scaffold that has been grown in the osteogenic medium (basal medium with 50 µg ascorbic acid, 1 µM dexamethasone and 10 µM β-glycerophosphate). The scaffold was rinsed with 1× PBS and added 0.1% Triton-X solution, then freeze–thawed three times. Afterward, substrate solution was measured using mouse OCN Elisa Kit. The resulted product was quantified by measuring absorbance at 450 nm on a microplate reader. The calcium concentration was calculated from a standard curve generated from serial dilution of calcium standard solution.

### Statistical analysis

The data were analyzed using IBM SPSS Statistics Standard 29.0.0.0 (SPSS, Chicago, IL, USA). The physicochemical characterization results were analyzed using descriptive analysis. For quantitative parameters, the normality of distribution was assessed with the Shapiro–Wilk test, and the homogeneity of variances was determined with the Levene test. One-way ANOVA was used for parametric data, and results were presented as mean ± SD. For parametric data without homogeneity of variances, Welch's ANOVA and Games-Howell's *post hoc* analysis were performed, and the data were presented as mean ± SD. The *P* < 0.05 was considered statistically significant.

## Results

### Physicochemical characterization

#### SEM and scanning electron microscope with energy-dispersive X-ray analysis

**Figure 1.**
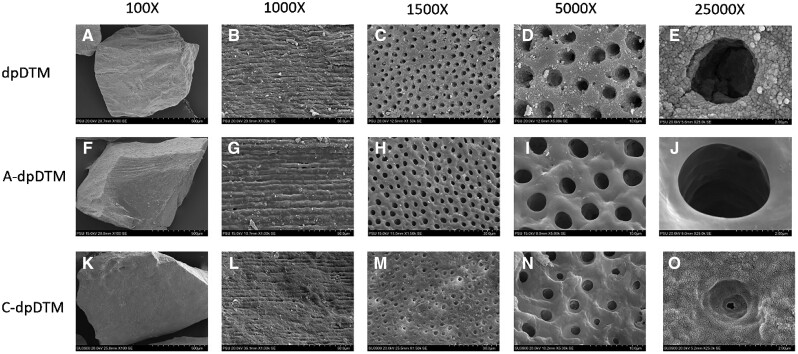
SEM images at magnification 100×, 1000×, 1500×, 5000× and 25 000× of dpDTM (**A**–**E**), A-dpDTM (**F**–**J**), and C-dpDTM (**K**–**O**).

**Figure 2.**
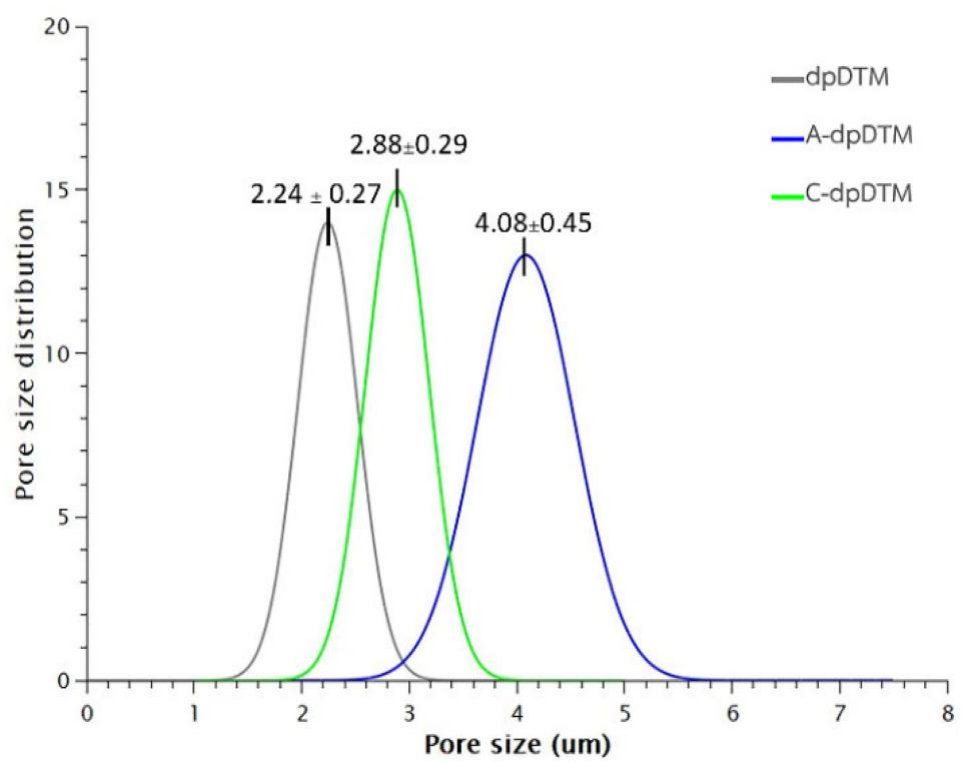
The pore size distribution and mean pore size of dpDTM, A-dpDTM, and C-dpDTM.

The surface morphology and microstructure of dpDTM, A-dpDTM, and C-dpDTM at 100×, 1000×, 1500×, 5000×, and 25 000× magnifications were depicted in Figure 1. The surface morphology of the dpDTM group demonstrated irregular surface and small dentinal tubules size with micro-cracks (Figure 1A–E). The surface morphology of the A-dpDTM group demonstrated larger opened dentinal tubules size and more irregular surface than dpDTM (Figure 1F–J). The surface morphology of the C-dpDTM group demonstrated irregular surface and dentinal tubules that were covered with collagen (Figure 1K–O). The mean pore size of A-dpDTM (4.08 ± 0.45 µm) was statistically significant higher than that of dpDTM (2.24 ± 0.27 µm, *P* = 0.000) and C-dpDTM (2.88 ± 0.29 µm, *P* = 0.000) as shown in Figure 2.

**Figure 3.**
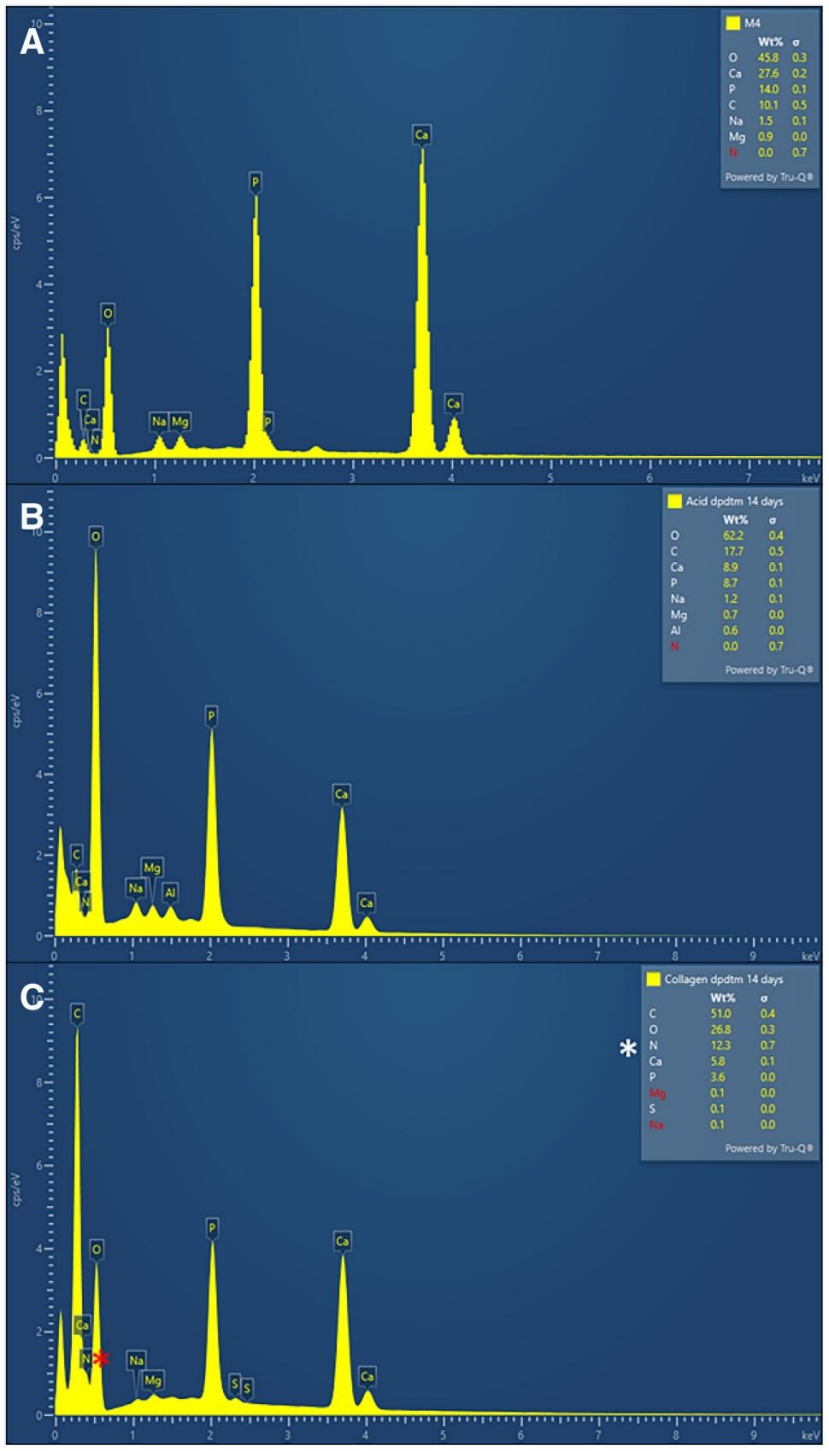
EDX spectrum of dpDTM (**A**), A-dpDTM (**B**), and C-dpDTM (**C**). The N (nitrogen) spectrum was only detected in C-dpDTM as indicated as *.

The EDX spectrum of dpDTM, A-dpDTM, and C-dpDTM was represented in Figure 3. The EDX spectrum of all samples detected main elements such as Ca (calcium), P (phosphate), O (oxygen), and C (carbon) except in the C-dpDTM, which represented the N (nitrogen) spectrum amount 12.3%wt (Figure 3C).

#### BET analysis

**Figure 4.**
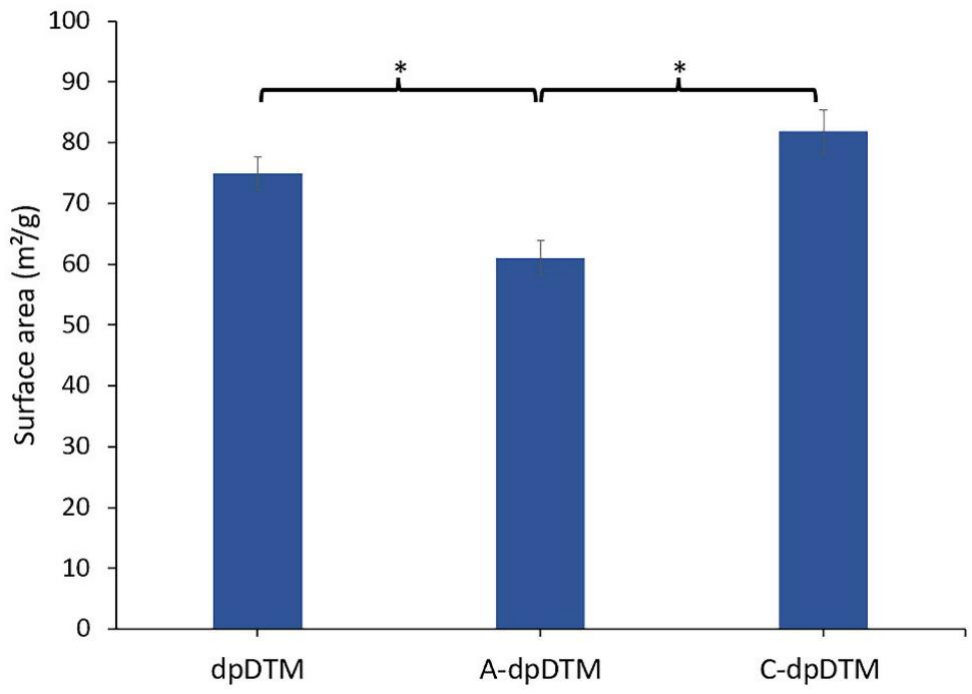
Surface area of the dpDTM, A-dpDTM, and C-dpDTM. *Significant difference between group at *P* < 0.05.

The surface area of A-dpDTM (61.01 ± 3.83%) was significantly lower than that of dpDTM (74.89 ± 2.72%, *P* = 0.039) and C-dpDTM (81.81 ± 3.49%, *P* = 0.013) as shown in Figure 4.

#### XRF and FTIR

According to XRF analysis, the major elements of all samples were calcium (Ca) and phosphorus (P), whereas the minor elements were sodium (Na), magnesium (Mg), chloride (Cl), silicon (Si), zinc (Zn), and strontium (Sr). The calcium and phosphate concentrations of the sample groups ranged from 32 to 36 wt% and 14 to 17 wt%, respectively. The Ca/P molar ratio of the sample in all groups was 1.67.

**Figure 5.**
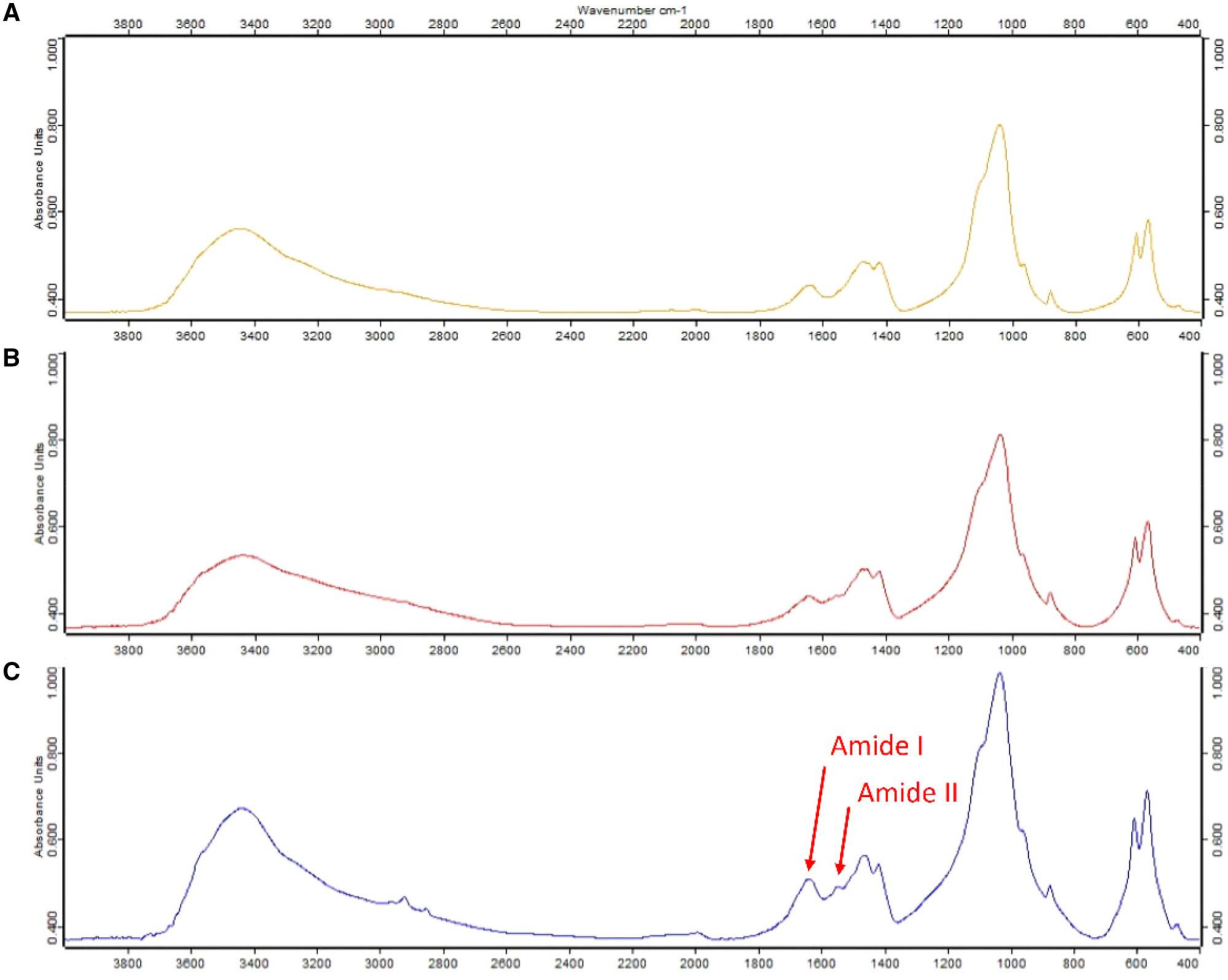
Absorbance FTIR spectra of dpDTM (**A**), A-dpDTM (**B**), and C-dpDTM (**C**).

The FTIR spectra of all groups were shown in Figure 5. The results of all groups showed characteristic absorption peaks of HA. There were peaks of the hydroxyl group at 3400–3500 cm^−1^ and the ${\text{PO}}_{4}^{3-}$ bands at 1031–1036, 604 cm^−1^. In addition, the results of the dpDTM and A-dpDTM groups demonstrated a lower amide I band, as shown in the lower peak at 1653 cm^−1^, and an absent amide II band at 1546 cm^−1^. This indicates that there was no remaining protein in the dpDTM and A-dpDTM groups. In contrast to dpDTM and A-dpDTM groups, the FTIR spectra of C-dpDTM groups demonstrated a higher amide I band and an amide II band.

#### XRD and fluorescence microscope

**Figure 6.**
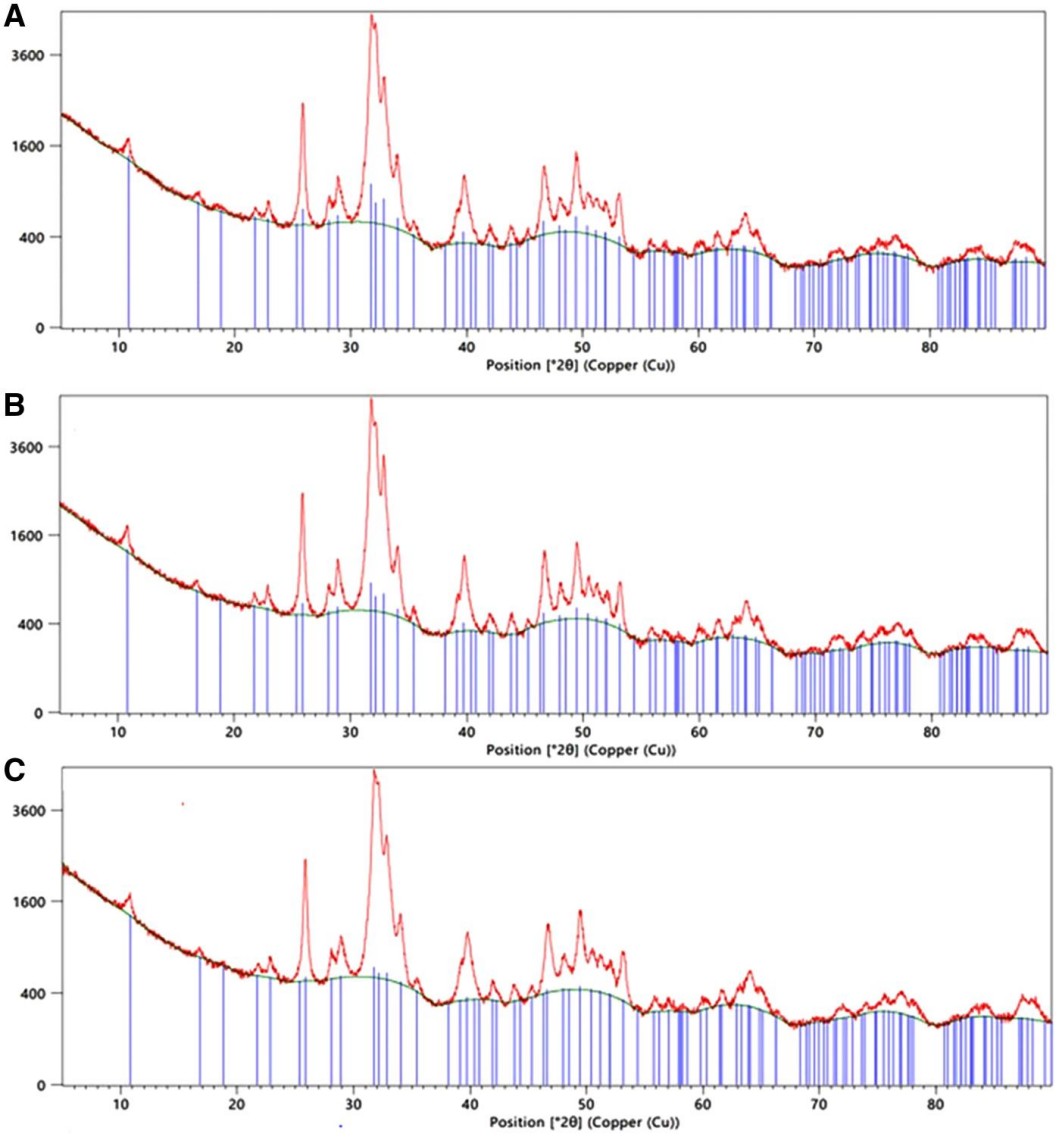
XRD patterns of dpDTM (A), A-dpDTM (**B**), and C-dpDTM (**C**).

The XRD patterns of dpDTM, A-dpDTM, and C-dpDTM were shown in Figure 6. According to the results, there was a HA phase in all groups. The crystallinity percentages of dpDTM, A-dpDTM, and C-dpDTM were 58.57±1.41%, 67.09±0.15%, 66.32±1.81%, and 65.90±0.25%, respectively. There was no significant difference in crystallinity between dpDTM, A-dpDTM, and C-dpDTM.

**Figure 7.**
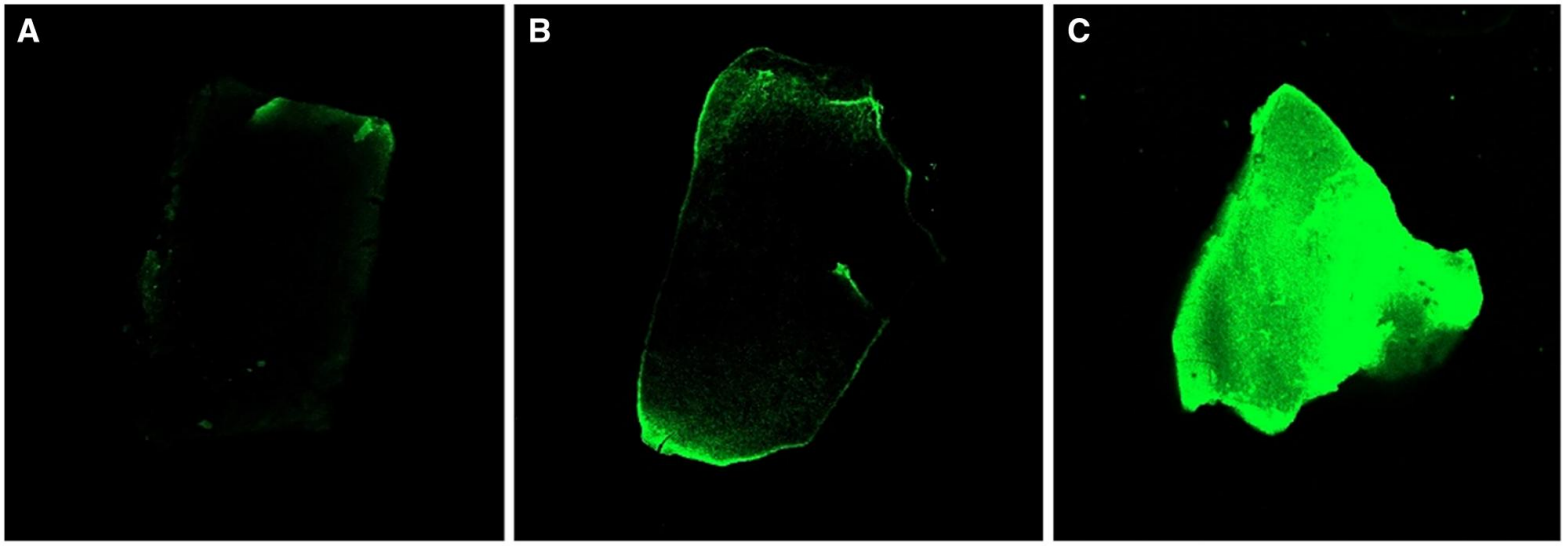
Fluorescence microscope image of dpDTM (**A**), A-dpDTM (**B**), and C-dpDTM (**C**).

The collagen immobilized on the surface of dpDTM was visualized under a fluorescent microscopy (Figure 7). The dpDTM (Figure 7A) and A-dpDTM (Figure 7B) were used as control and displayed no fluorescence after CBQCA derivatization. While fluorescence intensity was observed on the C-dpDTM (Figure 7C).

### *In vitro* degradation and collagen releasing

**Figure 8.**
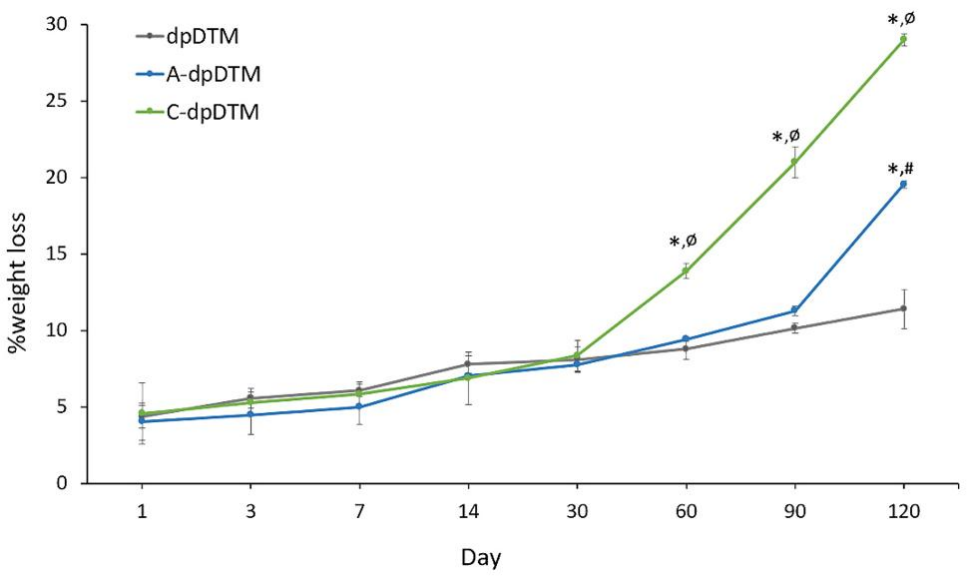
Percentage of weight loss as a function of degradation time in dpDTM (black), A-dpDTM (blue), and C-dpDTM (green) in SBF solution (pH 7.4) at different immersion period. *P < 0.05 statistically significant from dpDTM, ø*P* < 0.05 statistically significant from A-dpDTM, #*P* < 0.05 statistically significant from C-dpDTM.

The degradation in SBF solution (pH 7.4) in terms of percentage of weight loss was depicted in Figure 8. There was no significant difference in the weight loss among the three groups during the first 30 days of degradation. As the degradation time extended, the weight loss of dpDTM and A-dpDTM showed comparable results, while C-dpDTM exhibited a notably increased weight loss. After 60 days, the weight loss of C-dpDTM reached 13.89 ± 0.49%, which was significantly higher than dpDTM (8.79 ± 3.53%, *P* = 0.000) and A-dpDTM (9.45 ± 0.15% *P* = 0.000). After 120 days, the weight loss of C-dpDTM and A-dpDTM reached 28.99 ± 0.39% and 19.56 ± 0.24%, respectively, both significantly higher than dpDTM (11.42 ± 1.29%, *P* = 0.000).

**Figure 9.**
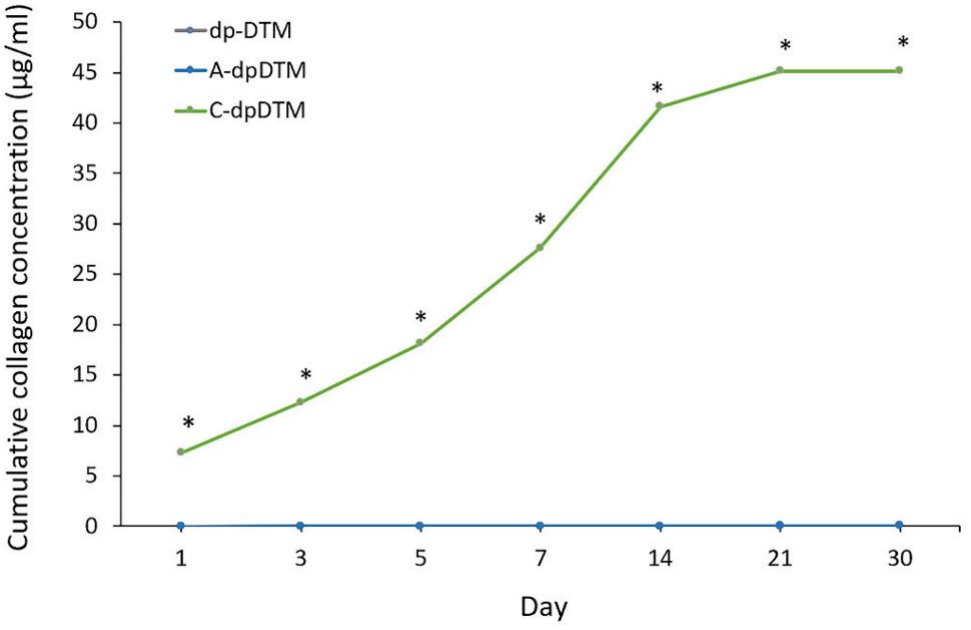
The cumulative collagen concentration of dpDTM, A-dpDTM, and C-dpDTM. *P < 0.05 statistically significant from dpDTM and A-dpDTM.

The cumulative collagen concentrations of dpDTM, A-dpDTM, and C-dpDTM in SBF up to 28 days were demonstrated in Figure 9. The C-dpDTM showed an initial release of collagen concentration on Day 1 and a burst release on Day 7, followed by a plateau on Day 21. The cumulative collagen concentration of C-dpDTM on Day 30 was 45.15 µg/ml. In addition, there was no release of collagen concentration in both dpDTM and A-dpDTM at all time points. It was noted that the cumulative collagen concentration of C-dpDTM was significantly higher than that of dpDTM and A-dpDTM at all time points (*P* < 0.05).

### Bioactivity characterization

#### Cell morphology

**Figure 10:**
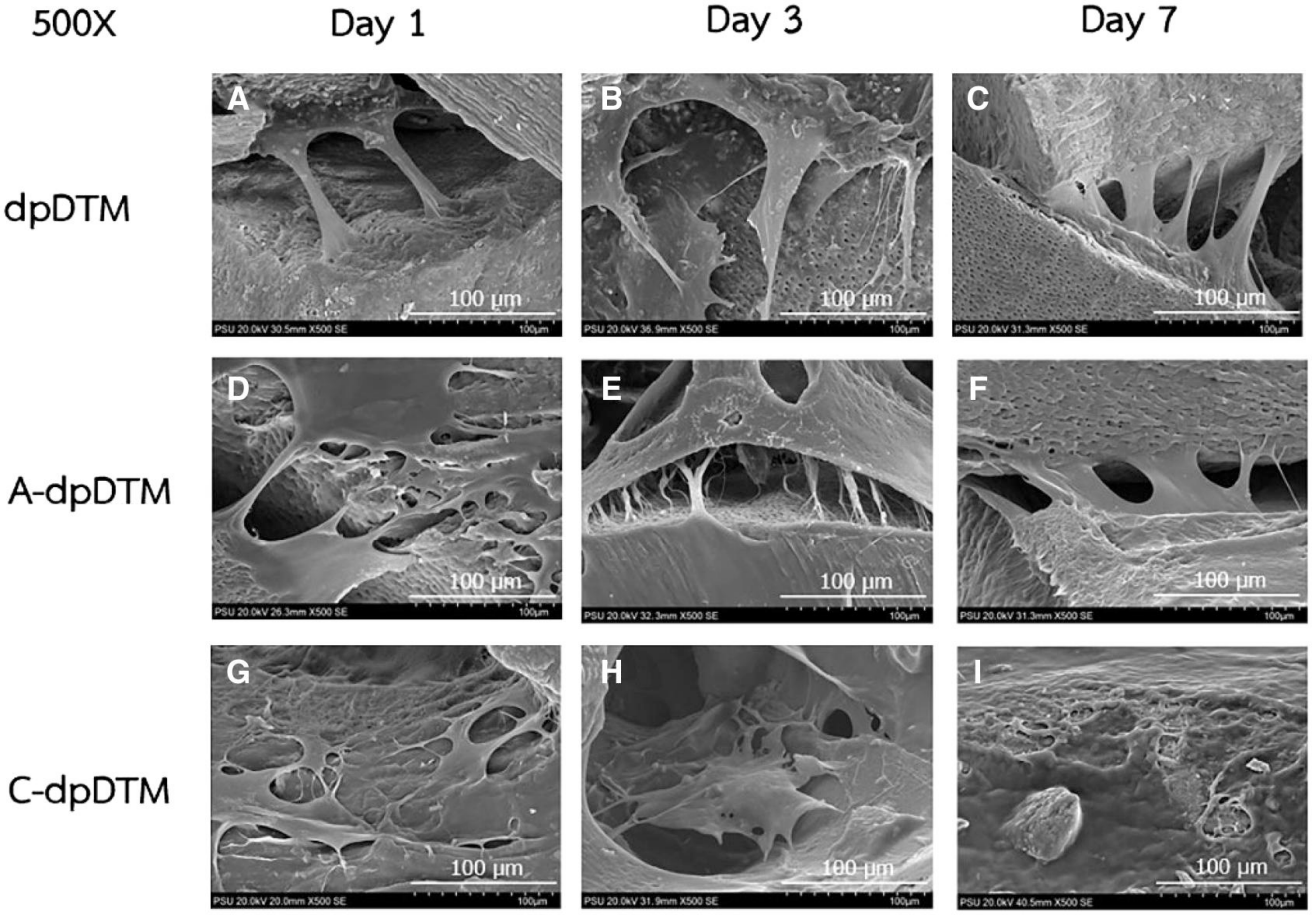
.SEM image of cell morphology and cell attachment on scaffolds of dpDTM (**A**–**C**), A-dpDTM (**D**–**F**), and C-dpDTM (**G**–**I**) on Days 1, 3, and 7 at magnification 500×.

Osteoblasts adhesion on the scaffold was observed by SEM on Days 1, 3, and 7. In dpDTM group, the morphology of cell attachment on Day 1 demonstrated a large flat shape and propagated on sample surface with an extended pseudopodia periphery through the dentinal tubules (Figure 10A). In A-dpDTM (Figure 10D) and C-dpDTM (Figure 10G), the cells spread on the surface of the samples and were more flattened, thicker, and showed distinct filopodia. On Days 3 and 7, it has been discovered that the cells proliferated effectively on the scaffold surfaces and produced multilayers, especially C-dpDTM, which at Day 7 established dense cell sheets covering the surfaces (Figure 10I).

#### Cell viability

**Figure 11.**
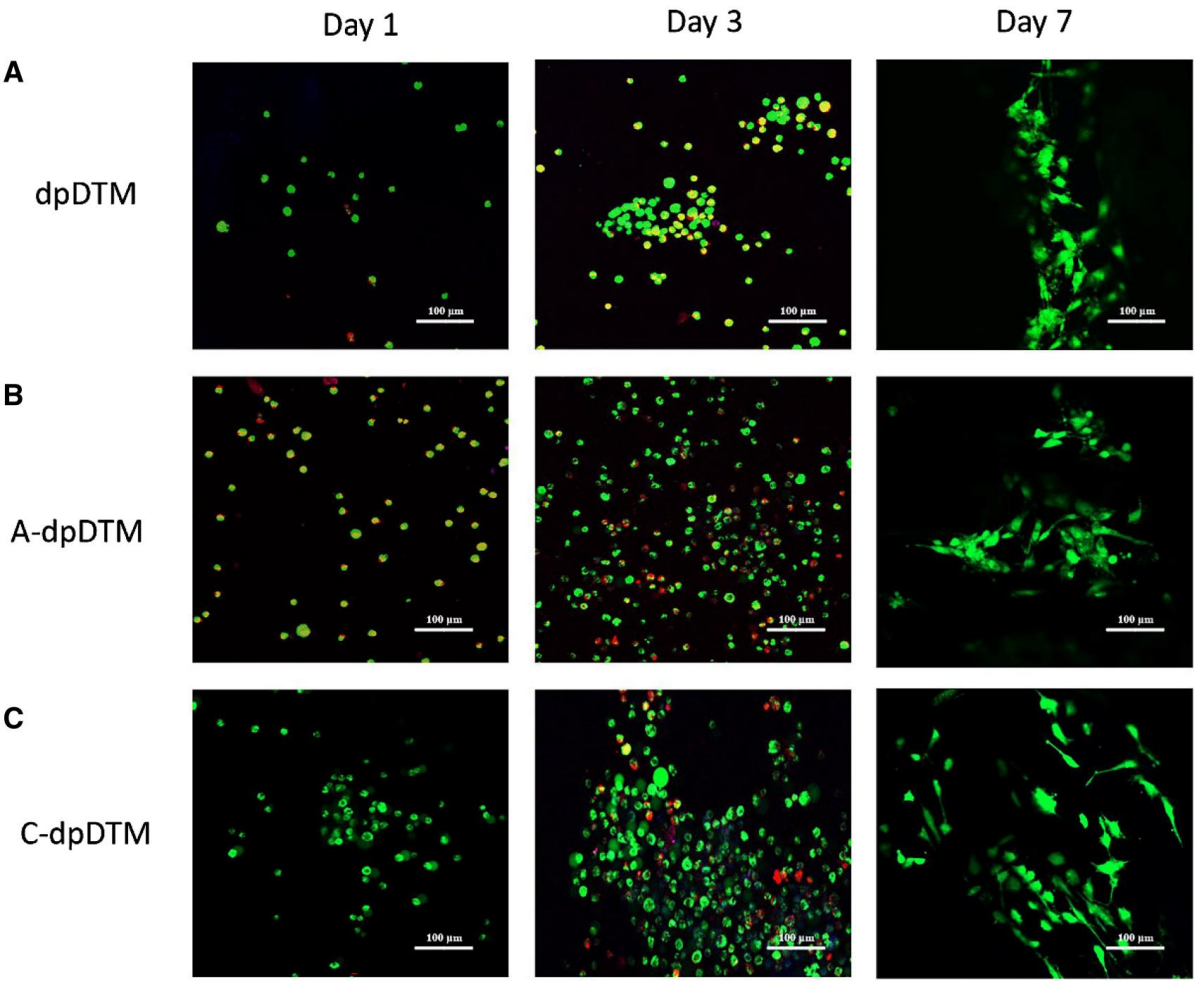
Confocal images of cell viability of dpDTM (**A**), A-dpDTM (**B**), and C-dpDTM (**C**). Green-live cells; red-dead cells; scale bar 100 μm.

Live and dead cell cytotoxicity was detected by CLFM on Days 1, 3, and 7 as shown in Figure 11. The cell survival rate of the dpDTM, A-dpDTM, and C-dpDTM were 61.58 ± 1.88%, 59.11 ± 7.24%, and 79.85 ± 4.44% respectively, on Day 1 (*P* = 0.004). As the culture time extended, the survival rates increased, reaching 88.87 ± 5.01%, 91.34 ± 1.97%, and 99.38 ± 0.12%, respectively, on Day 7 (*P* = 0.014).

**Figure 12.**
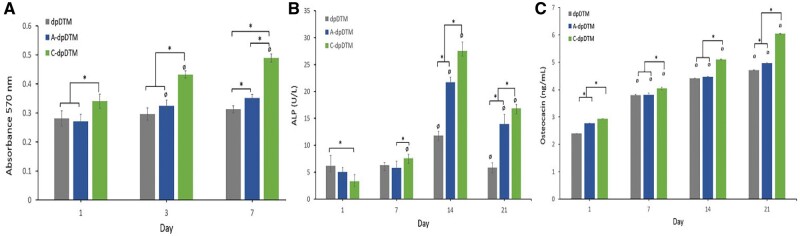
(**A**) The quantification of cell viability, (**B**) ALP activity, and (**C**) osteocalcin activity of dpDTM, A-dpDTM, and C-dpDTM. *Significant difference between the groups at *P* < 0.05, Ø significant difference with preceding time point at *P* < 0.05.

The quantification of the cell viability of dpDTM, A-dpDTM, and C-dpDTM on Days 1, 3, and 7 had been shown in Figure 12A. From Day 1 to Day 7, the cell proliferation of the MC3T3E1 osteoblast cell line increased gradually and reached its peak on Day 7 in all groups. The C-dpDTM had the highest cell proliferation and a significant difference from the other groups on Days 1, 3, and 7 (*P* < 0.05).

#### Alkaline phosphatase assay

The ALP activities of the cells were detected on Days 1, 7, 14, and 21 to determine the early stage of osteoblast differentiation. As shown in Figure 12B, The maximum peak of ALP was observed in all groups on Day 14, then decreased on Day 21. Moreover, the ALP levels of all groups showed a statistically significant difference from other time points at *P* < 0.05 on Day 14. The C-dpDTM had a statistically significant difference (*P* < 0.05) in higher ALP than dpDTM and A-dpDTM on Days 14 and 21.

#### Osteocalcin assay

The OCN activity was evaluated on Days 1, 7, 14, and 21, as shown in Figure 12C. The OCN was slightly increased from Day 1 until reaching its maximum peak on Day 21 in all groups. Each group had a statistically significant difference in OCN level between the different time points at *P* < 0.05. Moreover, the C-dpDTM had a statistically significant difference in higher OCN activity than dpDTM and A-dpDTM on Days 1, 7, 14, and 21 at *P* < 0.05.

## Discussion

In bone engineering scaffolds, surface characteristics play a crucial role in facilitating cell adhesion and proliferation [35]. Surface modification is implemented to enhance the surface characteristics of materials. Acidic conditions enhance surface morphology by selectively removing material, resulting in the creation of a roughened texture, and altered properties. This process is valuable in applications where improved adhesion, surface interactions, or specific material characteristics are desired. Coating surface with collagen is undertaken to replicate the natural ECM environment. This process facilitates improved interactions with cells, promotes both cell and tissue adhesion, and demonstrates inherent biocompatibility. In the present study, acid-modification and collagen-modification processes were effectively employed to modify the surface of dpDTM.

The A-dpDTM was successfully fabricated by 37.5% phosphoric acid. SEM revealed that the A-dpDTM exhibited opened dentinal tubules, larger pore sizes, and more irregular surface compared to the dpDTM, attributed to its phosphoric acid properties. Matos *et al*. [36] reported that 37.5% phosphoric acid etching of dentin caused the removal of the smear layer, exposing the apertures of dentinal tubules. Physiocochemical characteristics demonstrated no acid etching remaining on A-dpDTM and presented no secondary phase transformation, only a HA phase of calcium phosphate. This implied the successful surface modification of dpDTM through acid etching.

Acid etching can influence the surface morphology of calcium phosphate ceramic bone substitutes. Since A-dpDTM showed increased surface roughness and increased pore size, it opened dentinal tubules, but A-dpDTM had significantly less surface area than dpDTM and C-dpDTM, which can explain the inverse relationship between specific surface area and pore size. As the pore size decreases, the specific surface area increases, and vice versa. This is because smaller pores have a larger surface area relative to their volume compared to larger pores. Surface area is affected by microporosity and particle size, which depend on the manufacturing process. Phosphoric acid etching involves the chemical dissolution of the surface of the material. This dissolution can selectively remove surface layers, resulting in changes to the overall surface structure, including the pores. Depending on the concentration, duration, and conditions of the etching process, phosphoric acid can widen existing pores in the calcium phosphate structure. This can lead to an increase in average pore size and an alteration of the pore size distribution. The results of this study are similar to the study by Doi *et al*. [37], in which the porosity of HA surface treated with phosphoric acid was compared to porous HA. The findings showed that the percentage of porosity in the phosphoric acid-treated porous HA (77.71±4.68%) was significantly higher than that in porous HA (75.31±1.62%). Moreover, Abe *et al*. [38] reported the surface roughness of 30% phosphoric acid etching on HA via SEM image. The result demonstrated 30% phosphoric acid etching on HA had more surface roughness than the untreated HA.

In addition, the C-dpDTM was successfully fabricated by 37.5% phosphoric acid etching and 0.5% collagen coating. C-dpDTM was developed following the acid-modified dpDTM, as the acid etching process created larger pore sizes in dentinal tubules. This enlargement facilitated a more effective interlocking of collagen with the A-dpDTM surface. Additionally, acid etching increased the surface roughness, enhancing the ability for protein adhesion, thereby facilitating the easy attachment of collagen. To confirm collagen coating on A-dpDTM, SEM demonstrated an irregular surface and dentinal tubules that were covered with collagen, indicating that collagen was successfully coated. In addition, the SEM-EDX analysis study confirmed that C-dpDTM presented nitrogen (12.3 wt%), which is not detected in any of the remaining materials. The result of the collagen immobilized on the surface was observed only in the C-dpDTM group via fluorescent microscopy. It implied that the collagen coating was a successful surface modification.

In this study, C-dpDTM affected surface morphology. BET analysis and SEM demonstrated that C-dpDTM had more surface area and surface roughness than dpDTM. A collagen coating on calcium phosphate ceramic could increase the surface area due to several factors. Firstly, collagen was a fibrous protein that could form a nanostructured network when coated on the ceramic surface [39]. This nanostructured matrix had a high surface-to-volume ratio, resulting in an increased surface area [39]. Secondly, collagen coating could enhance the formation of mineralized bone matrix by providing nucleation sites for new mineral growth.

To assess the durability of the collagen coating, this study examined the release of collagen, revealing its presence up to Day 30. The cumulative release of collagen of the C-dpDTM showed an initial release of collagen concentration on Day 1 and a burst release on Day 7, followed by a plateau on Day 21. Longer collagen release in bone substitution is advantageous for several reasons. It promotes enhanced bone formation by providing a sustained environment for bone-forming cells, leading to improved stability and integration of substitute materials with surrounding bone tissue. Slow release of collagen reduces inflammation and foreign body responses, ensuring the body's acceptance of the material. The rate of collagen release in calcium phosphate materials could vary depending on various factors, including the type of calcium phosphate used, the method of incorporating collagen, the presence of crosslinking agents, and the specific experimental conditions [40].

Collagen coatings might degrade over a few weeks. However, the degradation time can be adjusted by modifying the crosslinking method, collagen concentration, and other factors. Collagen crosslinking with EDC-NHS is a chemical modification technique used to enhance the stability and durability of collagen-based materials. Everaerts *et al*. [41] demonstrated that EDC/NHS crosslinking of collagen leads to the formation of ester crosslinks, which could improve the mechanical and stability properties of collagen matrices. Additionally, while crosslinking could decrease degradation, collagen hydrogels crosslinked with EDC/NHS may still remain susceptible to collagenase degradation [42]. Hum and Boccaccini [43] compared collagen-releasing coatings on bioactive glass-based scaffolds with and without EDC/NHS crosslinking. The crosslinking process in SBF over the following 28 days led to a further reduction in released collagen, from 32% to 26%. In this study, the outcomes demonstrated the continued presence of collagen coating on the C-dpDTM surface even on Day 30. This suggests that the collagen coating with EDC-NHS crosslinking contributes to a reduction in the degradation rate of C-dpDTM compared to A-dpDTM.

In the present study, the degradation rate of C-dpDTM was about 28.99 ± 0.39% at 120 days, which was significantly higher than dpDTM and A-dpDTM. The degradability of bone substitute materials was influenced by a combination of factors, including composition, porosity, phase composition, surface morphology, particle size, porosity, and the process of preparation [44, 45]. Collagen possesses hydrophilic properties that could enhance water absorption by coated scaffolds. Increased water content could accelerate the degradation process by facilitating ion exchange and hydrolysis reactions. Furthermore, *in vivo* context, collagen-coated calcium phosphate scaffolds could promote cellular attachment, viability, proliferation, and activity. The presence of collagen could attract and support the adhesion and growth of cells, contributing to faster degradation [46, 47]. The degradation rate of C-dpDTM implies that C-dpDTM is a slowly degradable HA, making it a suitable alternative for use as a bone graft substitution material in dental surgery. This includes procedures such as alveolar ridge preservation for delayed implant placement, alveolar ridge augmentation, and maxillary sinus augmentation.

The crystallinity percentages of dpDTM, A-dpDTM, and C-dpDTM were higher than mineral tooth due to thermal treatment from deproteinization process. Wang *et al*. [48] investigated the control of crystallinity in a calcium phosphate bone cement and found that higher crystallinity resulted in reduced solubility and slower degradation of the cement. This suggests that higher crystallinity can contribute to the long-term stability and durability of the bone substitute material. However, surface modification process with acid etching and collagen coating did not significantly change the crystallinity. This contrasts with the findings of Yamashita *et al*. [49], who explored the presence of collagen molecules on ceramic surfaces and highlighted the potential hindrance to the growth of well-defined crystalline structures. The molecular structure of collagen might interfere with the growth of the crystal lattice, consequently resulting in reduced crystallinity. Nevertheless, coating calcium phosphate ceramics with collagen can potentially affect their crystallinity, although the exact impact would depend on various factors including the coating process, collagen concentration, and the specific type of calcium phosphate ceramic being used [50].

The Ca/P ratio of human bone has been reported to be approximately 1.67 [51]. In the present study, the Ca/P ratio in all groups was 1.67. This ratio is a defining characteristic specific to HA, a mineral recognized as the predominant calcium phosphate phase present in the human body. Thus, it implied that only the HA phase was present in all samples, which was consistent with the XRD results. The presence of HA is noteworthy due to its remarkable biocompatibility, structural resemblance to natural bone, and excellent osteoconductive properties.

Surface modification with phosphoric acid etching altered the surface morphology via increasing the pore size of A-dpDTM compared to dpDTM, showing an effect on cell biology as a result of SEM, which revealed that cells were able to adhere and showed gradually increasing cell proliferation on the surface of A-dpDTM. The presence of pseudopodia in osteoblast cells implied that the surface topography of the materials was conducive to cellular attachment and functionality. This result was consistent with the outcome of the live and dead cell cytotoxicity assessment. It demonstrated that living osteoblast cells on A-dpDTM increased over time compared to dpDTM. Additionally, the cell proliferation assay using the PestoBlue^®^ method demonstrated a similar trend in both results. A-dpDTM exhibited a higher cell proliferation rate than dpDTM. It could be inferred that A-dpDTM has no negative effect on viability proliferation and presents favorable biocompatibility and non-toxicity. The larger pore size of A-dpDTM appeared to promote cell adhesion and cell proliferation, supported by the significantly greater cell number observed on Day 7. This study shown larger pore size and rough surface of A-dpDTM induced more cell adhesion and cell proliferation. Previous studies have demonstrated that a material's rough surface offers increased surface area, significantly influencing initial cell adhesion [52, 53]. Doi *et al*. [37] evaluated the surface characteristics and bone formation stimulation abilities of the phosphate-treated porous HA. In an *in vivo* study, bone formation was observed in the phosphate-treated porous HA group, with a significantly higher bone formation ratio compared to the porous HA group. Thus, it implied that phosphoric acid etching on calcium phosphate surfaces enhances cell adhesion and bone formation.

Comparing A-dpDTM and C-dpDTM, C-dpDTM demonstrated significantly higher cell proliferation rates than A-dpDTM. Collagen coating on composite scaffolds plays a pivotal role in enhancing osteoblast adhesion and attachment. The presence of collagen on the scaffold surface introduces specific cell adhesion motifs, such as the arginine-glycine-aspartic (RGD) sequence, which serve as recognition sites for osteoblasts, promoting their effective attachment. Additionally, the bioactive nature of collagen-coated scaffolds amplifies signaling cues that facilitate osteoblast adhesion and subsequent differentiation [21, 22]. Furthermore, the modification of scaffold surface properties through collagen coating ensures an improved topographical landscape, fostering optimal osteoblast attachment, spreading, and growth. The rough surface could be more favorable for osteoblast adhesion [54], proliferation, and differentiation than smooth ones [55]. Furthermore, the microroughness seemed to be suitable for osteoblast differentiation [56]. Collectively, these mechanisms synergistically contribute to the enhanced osteoblast adhesion and attachment observed with collagen-coated composite scaffolds. Therefore, the modified surface morphology and chemical composition together create the overall interaction between cells and materials.

Due to the microporosity within the typical of dentin, less than 5 μm., provide a greater surface area which favorable protein adhesion and cell attachment on scaffold [57, 58] the inter-particle spacing indicates the osteoblast migration and proliferation. The particle sizes of about 380 μm in diameter would yield this minimal dimension of interparticular space [59]. The dpDTM and its modification were prepared with irregular shape and controlling size (500–1000 μm) which would yield this dimension of interparticulate space. The intrinsic microporosity and engineered macroporosity of these scaffolds provide the appropriate microenvironment and space for osteoblast interaction, adhesion, proliferation, and migration throughout the construct.

Both A-dpDTM and C-dpDTM groups exhibited notably higher ALP and OCN activity than dpDTM, suggesting enhanced early and late osteoblastic differentiation. Comparing A-dpDTM and C-dpDTM, the C-dpDTM displayed significantly higher ALP and OCN activity. Consequently, C-dpDTM not only improved adhesion and proliferation but also more effectively promoted osteoblastic differentiation compared to dpDTM and A-dpDTM. This confirms the biocompatibility of C-dpDTM and its potential to enhance osteoblast cell proliferation and differentiation.

Thus, both A-dpDTM and C-dpDTM are considered biocompatible and capable of promoting the proliferation and differentiation of osteoblast cells *in vitro*. Although *in vitro* cell biocompatibility tests are convenient, short time, and cost-effective for biomaterial screening, they lack the natural cellular environment and interactions, and the controlled culture conditions can affect cell growth and differentiation. Thus, further *in vivo* studies are necessary to validate the tissue biocompatibility and bone regeneration potential of surface-modified dpDTM.

## Conclusions

The development of natural bioactive bone scaffolds that emulate the structure and biological characteristics of native tissue is a fundamental requirement for addressing bone defects through tissue engineering. In this study, we successfully fabricated enhanced bioactive tooth scaffolds using an acid and collagen modification approach. The original dpDTM structure offers mechanical support, while in A-dpDTM, the acid treatment enhances surface properties, and C-dpDTM collagen ensures biomimetic and bioactive stimuli. These results hold promising potential for applications in bioengineered bone restoration.
